# Study on the Ecotoxic Effects of Uranium and Heavy Metal Elements in Soils of a Uranium Mining Area in Northern Guangdong

**DOI:** 10.3390/toxics11020097

**Published:** 2023-01-20

**Authors:** Zehui Zhang, Zhenping Tang, Yong Liu, Haiyang He, Zhixin Guo, Peng Feng, Liang Chen, Qinglin Sui

**Affiliations:** 1 School of Resource Environment and Safety Engineering, University of South China, Hengyang 421001, China; 2 Hunan Key Laboratory of Rare Metal Minerals Exploitation and Geological Disposal of Wastes, Hengyang 421001, China; 3 Hunan Province Engineering Technology Research Centre of Uranium Tailings Treatment Technology, Hengyang 421001, China; 4 Hunan Provincial Mining Geotechnical Engineering Disaster Prediction and Control Engineering Technology Research Center, Hengyang 421001, China; 5 State Key Laboratory of Nuclear Resources and Environment (East China University of Technology), Nanchang 330013, China

**Keywords:** uranium mines, biology, ecotoxicity, pollution evaluation

## Abstract

To investigate the heavy metal contamination of soil in a uranium mining area in northern Guangdong, a physicochemical evaluation method was used to evaluate the contaminated soil near the pit and tailings pond of the uranium mining area, determine its heavy metal content and evaluate its ecological risk using the Nemerow integrated contamination index, ground accumulation index and potential ecological risk index. The results show that the average content of nine heavy metal elements in the soil of the uranium mining area exceeds the background value of red soil in Guangdong Province. Three pollution evaluation indices all indicate that Cd, As and U have serious pollution and high ecological risk, while the remaining elements are weakly polluted and the potential ecological risk of the six sampling sites all show very strong risk. On this basis, soil ecotoxicity was evaluated using ostracods (*Cypridopsis vidua* and *Heterocypris* sp.), *Vibrio fischeri* and *Vicia faba* L. Higher concentrations of heavy metals at individual sites (T1, T2, P2) resulted in higher mortality of ostracods, higher inhibition of Vibrio fischeri luminescence and a significant reduction in germination and pigmentation of broad beans. The results of the biotoxicity evaluation were consistent with the results of the physicochemical evaluation, allowing for a more direct and comprehensive evaluation of the ecotoxic effects of uranium and heavy metals in the mine soils.

## 1. Introduction

After more than 30 years of development of nuclear power, China has made remarkable achievements [1,2]. Uranium has been widely exploited as the main fuel for nuclear energy. [3]. Uranium mining brings significant economic and social benefits to society but, at the same time, brings inevitable pollution to the surrounding natural environment. Uranium, which is radioactive and chemically toxic, is one of the most critical pollutants from uranium mining. It is also accompanied by heavy metal pollution, such as Cd, Cu, Pb, Mn and Zn, which are often reflected in the soil, sediment and water medium [4,5,6,7,8]. Heavy metal pollution around uranium mines has become a hot spot of concern because heavy metals are difficult to degrade, accumulate and easily migrate to the human body through skin, breath and diet [9,10,11]. At present, the evaluation of mine pollution is usually physical and chemical monitoring and evaluation, mainly using the Nemerow index [12,13], geoaccumulation index [14,15,16], potential risk index [17,18,19,20], etc. Although these evaluation methods can clearly understand the specific content of each pollutant in the environment and its changes, they cannot directly reflect the toxic effects of pollutants on organisms. Therefore, it is difficult to directly, comprehensively and accurately reflect the actual pollution status of a mine by simply using physical and chemical evaluation methods alone.

Biological monitoring is commonly used to evaluate the genotoxicity and mutagenicity of contaminants in organisms. Vibrio fischeri luminescence is the basis of several biologic methods of toxicity detection systems, including monitoring chemical toxicity in sewage and soil [21,22,23]. Higher plant toxicological experiments (seed germination experiment, root elongation experiment and early seedling growth experiment) are commonly used to detect the toxicity of various environmental contaminants because of their high sensitivity [24,25,26], ease of operation and low cost; ostracods are important parts of the aquatic ecosystem and belong to the phylum crustaceans of arthropods [27]. They are recognized as good environmental indicator organisms around the world and are widely used in toxicity experiments to monitor and evaluate heavy metal pollution on account of several advantages they possess, such as sensitivity to pollutants, large numbers, rich species, wide distribution, low research costs, easy collection and laboratory culture [28,29,30,31]. Khangarot et al. [32] proposed that it is necessary to include ostracods in biological tests to detect the presence of heavy metal contamination in soil, sludge, sediment and aquatic systems. The biomonitoring methods above have been widely used for ecological risk evaluation of contaminated soil, water bodies, sediments and sludge in polluted irrigation areas, cities and rural areas, but there have been fewer studies on the use of such methods for ecological risk evaluation of soil in uranium mining areas. The continuous accumulation of heavy metals in the soil of mining areas can lead to a decrease in their fertility and the contamination of crops and groundwater, which directly or indirectly endangers human health. With the increasing concept of environmental protection, society demands more comprehensiveness and accuracy of environmental evaluation, and the combination of physical and chemical evaluation and biological evaluation is an effective way to meet this demand [33].

A uranium mine in northern Guangdong is a vital uranium resource production base in China, with more than 50 years of mining history, and its surrounding ecological environment has inevitably suffered some degree of damage. At present, ecological monitoring and evaluation of this uranium mining area are still infrequent, and biological evaluation has not been reported. Therefore, in this study, we collected surface soil samples near a uranium mining area in northern Guangdong, determined their uranium and heavy metal element contents and evaluated them on the basis of physicochemical analysis by using three different trophic levels of ostracods (*Cypridopsis vidua* and *Heterocypris* sp.), *Vibrio fischeri* and *Vicia faba* L. Organisms were evaluated for soil ecotoxicity, and the results of the biological evaluation were compared with physicochemical data to scientifically evaluate the ecotoxic effects of soil uranium and heavy metal elements in this uranium mining area.

## 2. Materials and Methods

### 2.1. Sample Collection and Processing

**Figure 1:**
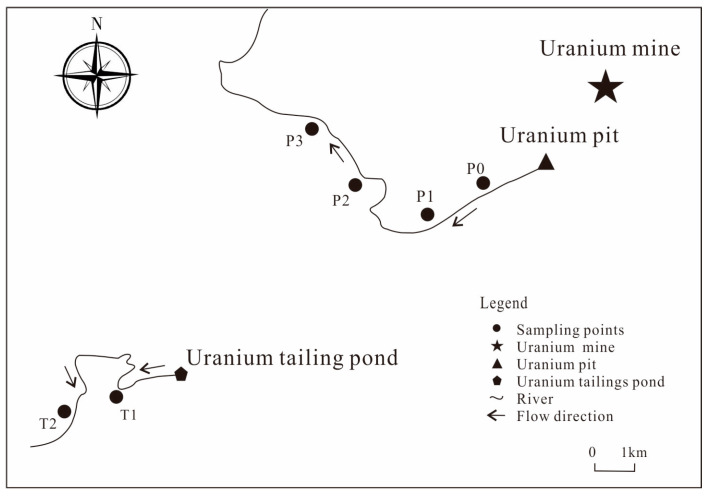
Sampling diagram of a uranium mining area in northern Guangdong.

Soil samples were collected in October 2020, including the uranium pit and tailings pond, with a total of 6 sampling points (Figure 1). Approximately 1 kg of soil was collected from the 0 ~ 20 cm surface layer, and the sample was sealed and stored in a sample bag after removing the debris. Soil samples were naturally dried to remove plant residues, gravel and other debris, ground through a 200-mesh sieve according to the quadratic method and placed in a desiccator for backup. The pH value was tested according to the specification (NY/T 1377-2007). The dissolved oxygen and conductivity were measured using a 1:5 ratio of a soil sample to water, shaking for 5 min, filtering the supernatant and testing with a multiparameter analyzer (Remag DZS-708-C). The soil organic matter content was measured and calculated using NY/T 1121.6-2006 for testing and calculation [34]; the basic parameters of the soil samples are shown in Table 1.

### 2.2. Determination of Heavy Metal Content in the Soil

The heavy metal content detection is based on the treatment method specified in the National Environmental Protection Standard of China, accurately weighing 0.1 g (±0.0002 g) of the dried soil sample in a polytetrafluoroethylene crucible, wetting it with a small drop of distilled water and then adding HCl, HNO_3_, HF and HClO_4_ in turn, placing it on an electric hot plate and heating it for digestion, cooling it to room temperature after the digestion is completed. On the electric hot plate, heat and drive out the acid until the internal solution is nearly dry, cool to room temperature, dissolve the endosome with deionized water and fix the solution into a 50 mL volumetric flask. The sample solution was filtered into a 10 mL centrifuge tube using a 0.22 μm aqueous filter membrane and refrigerated for measurement.

This study selected Mn, Ni, Cu, Cd, Pb, Zn, Cr, As and U, nine elements for testing, inductively coupled plasma mass spectrometer ICP-MS (X-Series11) for testing and analysis, completed in the National Defense Key Discipline Laboratory of Uranium Mining and Metallurgy Biotechnology, University of South China. The national standard soil GSS-25 was measured before determination of the samples to ensure the accuracy and reliability of the results. Each sample was tested three times, and Ge, In and Bi were selected as the standard internal elements to ensure the stability of the instrument and to obtain RSD < 5% for each element. The multielement standard solution was diluted with 5% HNO_3_ in steps of 0, 0.02, 0.04, 0.036, 0.08 and 0.1 mg·mL^−1^ for standard curve making.

### 2.3. Soil Contamination Physical and Chemical Evaluation Methods

#### 2.3.1. Nemerow Multifactor Index Method

The Nemerow composite index method is one of the most commonly used evaluation methods by domestic and foreign scholars; it was proposed by the American scholar Nemerow in 1977 and is widely used for soil pollution evaluation [35,36,37,38]. This method can highlight the impact of high concentrations of pollutants on soil environmental quality [39]. Its equation is as follows:

(1) $$P_{i}=C_{i}/S_{i}$$

(2) $$P_{N}=\sqrt{(P_{i\max}^{2}+P_{iave}^{2})/2}$$

where *P_i_* is the pollution index of heavy metal element *i*, *C_i_* is the measured concentration value of heavy metal element *i*, *S_i_* is the evaluation criterion of the heavy metal element, *P_N_* is the Nemerow pollution composite index, *P_imax_* is the maximum value of *P_i_* and *P_iave_* is the average value of each pollution element index. In this study, the background value of heavy metal elements in the zonal soil of Guangdong Province was chosen as the evaluation standard. The pollution level was divided into 5 levels according to the Nemerow integrated pollution index method, as shown in Table 2.

#### 2.3.2. Geoaccumulation Index

The geoaccumulation index method is an evaluation method proposed by the German scientist Müller in 1969, which considers not only the influence of anthropogenic pollution factors and environmental geochemistry on the background values but also the possible changes in background values due to natural diagenesis [40,41]. Meanwhile, to evaluate the combined effect of multiple heavy metal elements on the study area, Yao et al. [42] introduced the integrated geoaccumulation index (I_tot_), which is defined as the sum of all heavy metal ground accumulation indices (*I_geo_*) within an area. The geoaccumulation index was calculated as follows:

(3) $$I_{geo}=\log_{2}(\frac{C_{i}}{KB_{i}})$$

where *I_geo_* is the geoaccumulation index, *C_i_* is the measured content of heavy metal element *i*, *B_i_* is the geochemical background value of heavy metal element *i* and K is generally 1.5. The classification of the ground accumulation index evaluation results is shown in Table 3.

#### 2.3.3. Potential Ecological Risk Index Method

The potential ecological risk index method is a heavy metal pollution evaluation method proposed by the Swedish scientist Hakanson based on the nature and environmental behavior characteristics of heavy metals [43]. The method takes into account the ecological effects, environmental effects and toxicology of heavy metal pollutants and reflects the harm and impact of heavy metal elements on the ecological environment in a comprehensive manner [44,45,46]. Its calculation formula is as follows:

(4) $$C_{f}^{i}=\frac{C_{r}^{i}}{C_{n}^{i}}$$

(5) $$E_{r}^{i}=T_{r}^{i}\times C_{f}^{i}E_{r}^{i}=T_{r}^{i}$$

(6) $$RI=\sum_{i=1}^{n}E_{r}^{i}$$

where *C_f_^i^* is the pollution index of element *i*, where *C_r_^i^* is the measured content of element *i* and *C_n_^i^* is the background value of element *i*; *E_r_^i^* is the potential ecological risk of element *i* at the same point, where *T_r_^i^* is the toxicity response coefficient of element *i* and *RI* is the combined potential ecological risk of multiple elements at a sample point. The specific risk evaluation levels of the method are shown in Table 4.

### 2.4. Methods for the Biological Evaluation of Soil Contamination

#### 2.4.1. Methods for Evaluating Ostracod Toxicity

The experimental organisms were *Cypridopsis vidua* and *Heterocypris* sp., both of which were cultured for a long time in the laboratory (temperature 25 °C, light-dark ratio 16 h:8 h, pH 7.5 ± 0.2, dissolved oxygen > 5 mg/L), precultured for one week before the experiment and fed 24 h before the start of the formal experiment using the 4-day direct exposure acute toxicity test method. The test was performed on adult individuals of similar size and vigor and the solution temperature, pH, conductivity and dissolved oxygen content were measured before and after the experiment. There were 3 parallel groups for each sample, and a blank control group was set up. Ten worms in each treatment group were placed in beakers with 40 mL of sample extract (1:10 mixture of soil sample and distilled water, shaken at 120 r/min for 8 h, centrifuged and the supernatant filtered), and the number of dead worms in each treatment group was observed and recorded at 24, 48, 72 and 96 h. A time-point mortality curve was obtained. A total of 190 worms in 19 treatment groups were used for this experiment, and death was defined as the absence of life activity within 15 s of shaking the beaker.

#### 2.4.2. Methods for the Evaluation of Luminescent Bacteria

Preparation of *Vibrio fischeri* lyophilized powder: *Vibrio fischeri* lyophilized powder was purchased from Zhejiang Tocos Biotechnology Co. *Vibrio fischeri* lyophilized powder was prepared according to the ISO–11348 standard [47]. According to the bacterial concentration of OD 600 = 1, protective agent was added to 2 mL sterile EP tubules, 100 μL was added to each tube and the tubes were freeze-dried in a vacuum and stored at −20 °C under light for later use.

Recovery of strain and preparation of bacterial solution: One part of Vibrio fischeri lyophilized powder and one part of recovery solution were combined and equilibrated at room temperature for 15 min. Two milliliters of recovery solution was injected into the lyophilized powder reagent bottle and left for 10 min. After the recovery was completed, Vibrio fischeri was diluted with 2% sodium chloride solution at a ratio of 1:4 to obtain bacterial dilution solution.

Soil sample detection: The bacterial dilution was mixed with the sample to be tested at a ratio of 1:90, and a blank group was set up with a reaction time of 15 min. The luminescence intensity was measured with a bioluminescence detector (Glomax 20/20 bioluminescence detector), and the relative luminescence intensity (relative luminescence intensity = sample luminescence intensity/blank luminescence intensity) was calculated. The luminescence inhibition rate = 1−relative luminescence intensity. The luminescence inhibition rate =1−relative luminescence intensity.

#### 2.4.3. Methods for Evaluating the Toxicity of Fava Beans

Seedling growth assay: Whole, uniform broad bean seeds were sterilized with 0.5% NaClO solution for 20 min, rinsed and soaked in distilled water for 1 day. Place them in a Petri dish lined with double–layer filter paper, add 10 mL of each treatment solution (distilled water was used as the control), add 8 broad bean seeds per treatment group, set up three parallel samples for each concentration treatment and incubate them at 25 °C for 7 days under no light conditions. After 3 days of germination, the number of seeds germinating was observed and recorded on a daily basis (germination was judged by the length of the germ reaching half of the seeds), germination rate = the number of seeds germinating at 7 days/number of seeds tested × 100%.

The chlorophyll content was determined by the colorimetric method. On the 7th day of incubation, 0.1 g of leaves were taken from each of the three parallel samples of each concentration in the same position, placed in a centrifuge tube, 10 mL of 80% acetone was added and the absorbance values were measured at 663 mm, 646 mm and 470 mm in the dark for 40 h (after the leaves had whitened), with 80% acetone as the control.

The calculation formula is:

Chlorophyll a concentration (mg/L) = 12.21 × OD663−2.81 × OD646

Chlorophyll b concentration (mg/L) = 20.13 × OD646−5.03 × OD663

Carotenoid concentration (mg/L) = (1000 × OD470−3.27 × Chlorophyll a concentration−1.0 × Chlorophyll b concentration)/229

Chlorophyll content (mg/gFW) = chlorophyll concentration × acetone volume of extract/leaf weight

## 3. Results and Discussion

### 3.1. Heavy Metal Elements and Uranium Content

The soil pH varied from 7.25 to 7.40, with a mean value of 7.32, which was weakly alkaline. The total amount of heavy metals in the soil samples was positively correlated with the conductivity; that is, the conductivity of the sample points with low heavy metal contents also decreased. The heavy metal content in this study was significantly controlled by the source of pollution, i.e., the closer to the source, the higher the heavy metal content of the samples in general, and the distance increased, the heavy metal content decreased, and the relationship with organic matter content was not significant.

The heavy metal content of the soil in the study area is shown in Table 5. Due to the missing screening values of Mn, Co and U elements in the “Soil Environmental Quality Agricultural Land Soil Pollution Risk Control Standard” [48], this study is not able to use these data for pollution assessment. Therefore, the background soil value of the province where the mining area is located is used as the evaluation basis. In addition, the soil around the sampling site is red, and the background value of red soil in Guangdong zonal soil is used as the basis of evaluation in this paper [49], but the background value of element U is missing. Therefore, element U refers to the background value of the Chinese soil environment [50]. Analysis of the content of heavy elements and U in the soil at six sampling points around the mine showed that the content of As ranged from 605 to1234 mg/kg, followed by Mn at 247 to 713 mg/kg and U at 20.2 to 43.5 mg/kg. All three elements were at their highest levels at T1. At each point site, tailings pond T1 and pit P2 have a higher content of each element compared to the other sites. According to the results of the physicochemical parameters, the average content of heavy metals in the soil of a uranium mining area in northern Guangdong is As > Mn > Zn > Pb > Co > U > Ni > Cd in order of magnitude. Compared to the background values of the zonal soil environment of Guangdong Province, the mean values of the nine elements exceeded their background values, with Mn 2.9 times the background value, Co 15.6 times, Ni 2 times, Cu 1.7 times, Zn 3.2 times, As 90 times, Cd 241 times, Pb 2.1 times and U 8.1 times. The coefficient of variation of the content of a certain heavy metal reflects the variability of its distribution and pollution level in a certain area. The larger the value is, the greater the disturbance of the environment by human activities [51]. The coefficients of variation of U and Zn were 39.34% and 30.21%, respectively, which were of medium variability; the coefficients of variation of Ni, Cu and Pb were 27.86%, 20.64% and 21.66%, respectively, with slight differences in distribution.

### 3.2. Physical and Chemical Evaluation of Soil Contamination

#### 3.2.1. Evaluation of the Nemerow Composite Pollution Index Method

The Nemerow Integrated Pollution Index method evaluates both the average and maximum values of individual pollution indices, highlighting the impact and effect of the more polluting pollutants on the environment and is able to comprehensively reflect the overall quality of the soil environment; the evaluation results are shown in Table 6. The order of the single factor pollution index is Cd > As > Co > U > Mn > Zn > Ni > Pb > Cu. Cd, As, Co and U are at a serious pollution level and contribute more to environmental pollution, Zn is at a moderate pollution level, and Mn, Ni, Pb and Cu are at a low pollution level. The *P_N_* ranged from 102.97 to 213.66, with an average of 171.80. The lowest and highest values were found at the P1 point and the highest at the T1 point. The order of the Nemerow comprehensive pollution index from large to small was T1 > T2 > P2 > P0 > P3 > P1. Overall, the six samples in the study area were all severely polluted, and the main contributing factor was Cd. The average value of the single pollution index was 239.56.

#### 3.2.2. Evaluation of the Geoaccumulation Index

**Figure 2:**
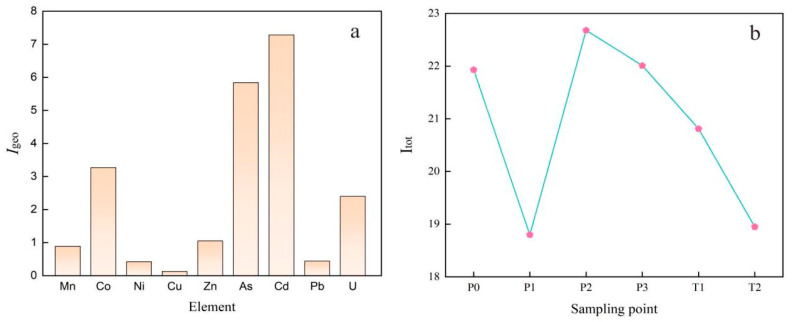
Mean plot of soil uranium and heavy metal accumulation index (**a**) and sum plot (**b**) of a uranium mining area in northern Guangdong.

The results and grading of heavy metal elements and uranium accumulation indices of the soils in the uranium mining area are shown in Table 7. Nine elements were contaminated to different degrees at different sites, and the accumulation indices of each element varied greatly at the same sites. The ranking of each element from highest to lowest was Cd > As > Co > U > Zn > Mn > Pb > Ni > Cu (Figure 2a). The results of the single-element geoaccumulation index (*I_geo_*) evaluation show that Cd and As are severely polluted, Co and U are moderately polluted, Zn is moderately polluted, and Mn, Pb, Ni and Cu are lightly polluted. The results of the Integrated Geoaccumulation Index (I_tot_) (Figure 2b) indicate that the mine site is extremely polluted with heavy metals at level 6, while the main pollutants in the mine site are Cd and As according to the Igeo rating, which is basically consistent with the results of the Nemerow Integrated Pollution Index.

The results and grading of heavy metal elements and uranium accumulation indices of the soils in the uranium mining area are shown in Table 7. Nine elements were contaminated to different degrees at different sites and the accumulation indices of each element varied greatly at the same sites. The ranking of each element from highest to lowest was Cd > As > Co > U > Zn > Mn > Pb > Ni > Cu (Figure 2a). The results of the single-element geoaccumulation index (*I*_geo_) evaluation show that Cd and As are severely polluted, Co and U are moderately polluted, Zn is moderately polluted and Mn, Pb, Ni and Cu are lightly polluted. The results of the Integrated Geoaccumulation Index (I_tot_) (Figure 2b) indicate that the mine site is extremely polluted with heavy metals at level 6, while the main pollutants in the mine site are Cd and As according to the Igeo rating, which is basically consistent with the results of the Nemerow Integrated Pollution Index.

#### 3.2.3. Evaluation of the Potential Ecological Risk Index Method

The evaluation of the potential ecological risk index of soil in a uranium mining area in northern Guangdong showed (Table 8) that the mean ecological risk Eri values of soil heavy metal elements ranged from 2.99 to 7186.84, with large differences among elements. Among them, Cd has the highest ecological risk, which is very high. The risk value of Cd at various points is also significantly higher than that of other elements, ranging from 4301.03 to 8933.82, with a large difference in distribution. The reasons for this result include two main aspects, first due to its high content and second related to the toxicity response factor *T_r_^i^*. Hakanson has set toxicity factors based on the toxicity of different elements. The toxicity factors for the heavy metals involved in this paper are 1 (Mn, Zn), 5 (Co, Ni, Cu, Pb), 10 (As) and 30 (Cd) and the toxicity factor for radionuclide U is currently missing, but there is no doubt about the severity of its impact on the environment, which is both chemically and radiologically toxic. Therefore, this paper set the toxicity coefficient of U by referring to Cd element 30, which has the highest toxicity coefficient among the studied heavy metals. As and U showed high risk, and Co showed low risk. The evaluation results for the elements Mn, Ni, Cu, Zn and Pb showed no risk, with a small fluctuation range at each sampling point. The potential ecological risk index for heavy metal elements in the uranium mining area is Cd > As > U > Co > Pb > Ni > Cu > Zn > Mn in descending order. Each of the sampling sites had the highest combined potential risk index rating, showing a very high risk.

In comparison with the above two types of physical and chemical evaluation results, it was found that the results of the Nemerow integrated pollution index method, the evaluation of the geoaccumulation index and the potential ecological risk index method all indicated that Cd, As, Co and U were the main polluting elements in the study area and that their contributions to heavy metal pollution in the mine area were greater. The corresponding evaluation results often differ for each method due to their different evaluation perspectives, with the remaining five elements being minor pollutants in their respective evaluation systems.

Qin et al. [52] evaluated the ecological risk of decommissioned uranium mine soil. The results showed that the concentrations of the heavy soil metals Cd and As were above the limits of environmental quality standards, with Cd having the highest potential ecological risk, followed by As. Zhang et al. [53] investigated the soil contamination around uranium tailings ponds and found that the contamination was more serious within 0−1 km of the tailings ponds, mainly caused by Cd, As and U. The degree and risk of elemental contamination decreased with increasing distance from the tailings ponds. Therefore, Cd, As and U are the main contaminating elements in the soils of our study area and the pattern of change in the degree of contamination and risk level of the tailings ponds (T1 and T2) is consistent with the study by Zhang et al. [53]. Based on the above results, the average concentrations of Cd, As and U far exceeded their background values and all three elements had high concentrations even at low concentrations, with toxicity factors of 30 for Cd and U and 10 for As [54,55].

### 3.3. Biological Evaluation of Soil Contamination

#### 3.3.1. Evaluation of Ostracod Toxicity

**Figure 3:**
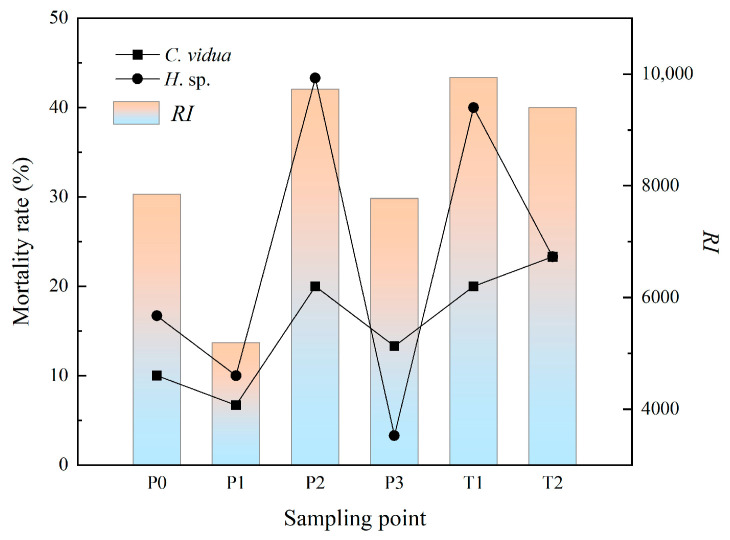
Relationship between potential ecological risk *RI* and Ostracod mortality.

The results of the ostracod toxicity evaluation are shown in Table 9. For *C. vidua*, the highest mortality rate of 23.3% was recorded at sample site T2, followed by sample sites T1 and P2 with a mortality rate of 20%, and the lowest mortality rate of 6.7% was recorded at sample site P1 at the pit entrance for a 96 h termination time exposure. For *H.* sp., the 96 h termination time exposure had the highest mortality rate of 43.3% at the pit P2 sample site, followed by the T1 sample site with 40% and the lowest mortality rate of 3.3% at pit P3. The mortality rates of P2, T1 and T2 at the termination time of the two biological experiments were higher than those of other samples, and the potential ecological risk index of these samples was higher (Figure 3). The potential ecological risk index of P1 and P3 was obviously low, and the mortality of ostracods was also low at the two sites. The difference between the potential ecological risk index of P0 and P3 was very small, and the mortality of the *C. vidua* endpoint was not significant at these two sites. Still, in *H.* sp., the mortality rate at P0 was significantly higher than that at P3, and the results of the two biological evaluations showed specific differences here. The above analysis indicated that the results of the ostracod assessment were consistent with the results of the potential ecological risk assessment.

#### 3.3.2. Toxicity Evaluation of Fava Beans

**Figure 4:**
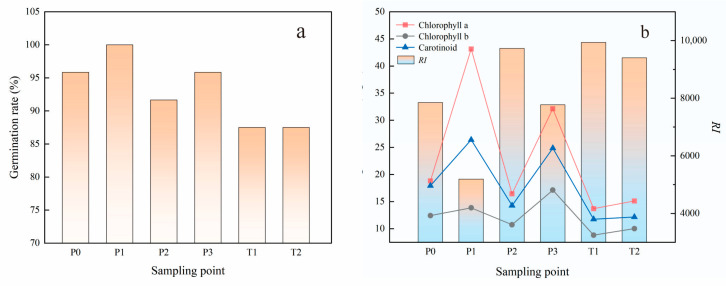
Relationship between germination rate (**a**), potential ecological risk *RI* value and pigment content of *Vicia faba* L. (**b**).

The effect of soil on Vicia faba L. seed germination differed at different points in the study area (Figure 4a). The germination rate of broad beans at T1, T2 and P2 was lower than that at the other sites, with 87.50% germination at T1 and T2, 91.67% germination at P2, 100% germination at P1 and 95.83% at the remaining sites. Heavy metals inhibit chlorophyll synthesis by inhibiting its activity. Comparing the pigment content of fava bean with the potential ecological risk index RI (Figure 4b), the results showed that the T1, T2, P2 and P0 values at risk were higher, the levels of broad bean seedling leaf chlorophyll a and chlorophyll b and carotenoid content decreased significantly, and the chlorophyll a and carotenoid content changed more significantly than chlorophyll b. The highest RI and lowest pigment content at T1 were consistent with the potential risk. The results of the evaluation were consistent with the results of the potential risk.

#### 3.3.3. Toxicity Evaluation of Luminescent Bacteria

**Figure 5:**
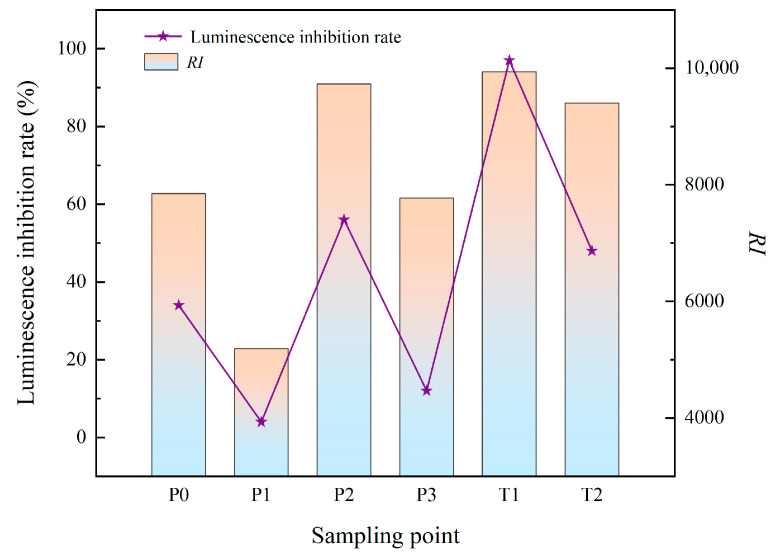
Diagram of the *RI* value of the potential ecological risk and luminescence inhibition rate of *Vibrio fischeri.*

*Vibrio fischeri* luminescence is highly susceptible to external conditions. When luminescent bacteria come into contact with toxic and harmful substances, they affect their physiological and metabolic processes and thus cause changes in luminescence intensity, which generally tends to diminish with increasing concentrations of toxic substances. The strongest luminescence inhibition was at T1 with 97% inhibition, followed by P2 and T2 with 56% and 48% inhibition, respectively, P0 with 34% luminescence inhibition, and P1 and P3 points with 4% and 12% inhibition, respectively. The luminescence inhibition rate was compared with the potential ecological risk index and analyzed (Figure 5). The luminescence inhibition rate was significantly higher at the higher potential risk index points (T1, P2, T2, P0), with the highest RI at T1 having the most significant luminescence inhibition and the luminescence inhibition rate at P1 and P3 being significantly lower.

Compared with the above biotoxicity test data, it was found that the mortality of ostracod individuals and the inhibition of luminescent bacteria were positively correlated with the degree of environmental pollution at the sample sites; the germination rate and pigment content of fava beans were negatively correlated with the degree of environmental pollution. The use of a single species in environmental assessment generally has limitations, and the combination of different nutrient levels can more accurately assess the environmental pollution level.

## 4. Conclusions

The main insights gained in this paper are as follows:

(1) The study area as a whole is at a high-risk level, with higher levels of contamination in tailings ponds than in pits. Cd, As and U are the main polluting elements in the study area. The degree of elemental contamination in the tailings ponds was negatively correlated with the distance from the tailings ponds.
(2) A combination of physical and chemical evaluation and biological evaluation is used to more directly and comprehensively reflect the characteristics of uranium and heavy metal contamination in mine soils.

## Figures and Tables

**Table 1:** Basic parameters of the soil samples.

Sampling Point	pH	Dissolved Oxygen (mg/L)	Electrical Conductivity (µS/cm)	Organic Matter (mg/kg)
P0	7.29	4.47	94.8	8200
P1	7.25	4.77	58.7	13,600
P2	7.37	3.54	55.1	6250
P3	7.26	4.15	45.5	17,000
T1	7.40	3.78	136.7	4900
T2	7.34	5.06	127.3	9800
Average value	7.32	4.30	86.35	9958

**Table 2:** Nemerow comprehensive pollution index classification table.

Pollution Class	*P_i_*	*P_N_*	Pollution Level
1	*P_i_* ≤ 1	*P_N_* ≤ 0.7	Clean
2	1 < *P_i_* ≤ 2	0.7 < *P_N_* ≤ 1	Slight
3	2 < *P_i_* ≤ 3	1 < *P_N_* ≤ 2	Light pollution
4	3 < *P_i_* ≤ 5	2 < *P_N_* ≤ 3	Medium pollution
5	*P_i_* > 5	*P_N_* > 3	Severe pollution

**Table 3:** Grading standard was evaluated by the ground accumulation index method.

Pollution Class	*I*_geo_	Pollution Level
0	*I*_geo_ < 0	Clean
1	0 ≤ *I*_geo_ < 1	No pollution–slight pollution
2	1 ≤ *I*_geo_ < 2	Medium pollution
3	2 ≤ *I*_geo_ < 3	Medium pollution–Heavy pollution
4	3 ≤ *I*_geo_ < 4	Heavy pollution
5	4 ≤ *I*_geo_ < 5	Heavy pollution–very heavy pollution
6	*I*_geo_ ≥ 5	Very heavy pollution

**Table 4:** Potential risk index classification of the degree of pollution.

Pollution Level	*E_r_^i^*	*RI*
Low	*E_r_^i^* < 40	*RI* < 150
Moderate	40 ≤ *E_r_^i^* < 80	150 ≤ *RI* < 300
Considerable	80 ≤ *E_r_^i^* < 160	300 ≤ *RI* < 600
Heavy	160 ≤ *E_r_^i^* < 320	600 ≤ *RI* < 1200
Serious	*E_r_^i^* ≥ 320	*RI* ≥ 1200

**Table 5:** Content of uranium and heavy metals in the soil of a uranium mining area in northern Guangdong (mg/kg).

Projects	Heavy Metal Elements (mg/kg)									Total
	Mn	Co	Ni	Cu	Zn	As	Cd	Pb	U	
P0	328 ± 2	59.2 ± 1.7	29.4 ± 1.5	26.1 ± 1.2	190 ± 2	820 ± 2	7.64 ± 0.36	74.9 ± 1.8	30.3 ± 1.3	1565.90
P1	247 ± 1	45.5 ± 0.8	19.8 ± 0.9	22.9 ± 0.4	166 ± 1	605 ± 1	4.87 ± 0.15	56.7 ± 0.3	32.4 ± 1.2	1200.07
P2	325 ± 2	90.1 ± 2.3	32.9 ± 1.3	26.3 ± 1.2	161 ± 2	1113 ± 2	9.38 ± 0.33	78.5 ± 1.3	38.2 ± 1.2	1874.60
P3	281 ± 2	30.2 ± 0.5	23.0 ± 0.9	28.2 ± 1.3	160 ± 1	671 ± 3	7.28 ± 0.14	72.7 ± 1.5	20.2 ± 0.7	1303.49
T1	713 ± 1	94.6 ± 0.6	22.6 ± 0.2	18.1 ± 0.9	128 ± 1	1234 ± 2	10.51 ± 0.26	69.8 ± 0.4	43.5 ± 0.1	2333.41
T2	481 ± 3	83.4 ± 2.1	22.1 ± 0.3	21.8 ± 0.8	118 ± 3	1171 ± 3	9.27 ± 0.35	70.4 ± 2.0	35.1 ± 1.5	2011.78
Highest value	713	94.6	32.9	28.2	190	1234	10.51	78.5	43.5	2425.21
Lowest value	247	30.2	19.8	18.1	118	605	4.87	56.7	20.2	1119.87
Average	395.7	67.2	26.6	23.9	153.8	935.7	8.2	70.5	33.4	1715
Coefficient of variation	47.53%	50.23%	27.86%	20.64%	30.21%	45.80%	43.57%	21.66%	39.34%	-
Risk screening values	-	-	100	200	250	25	0.6	140	-	-
Soil background values	132.5	4.30	13.00	14.38	48.75	10.50	0.034	34.38	4.11	-

Note: “-” means not specified.

**Table 6:** Statistical table of the improved Nemerow comprehensive pollution index for uranium and heavy metals in the soil of a uranium mining area in northern Guangdong.

Sampling Points	Mn	Co	Ni	Cu	Zn	As	Cd	Pb	U	*P*_N_
P0	2.48	13.76	2.26	1.82	3.89	78.14	224.71	2.18	7.37	161.08
P1	1.86	10.59	1.53	1.59	3.41	57.61	143.37	1.65	7.89	102.97
P2	2.45	20.94	2.53	1.83	3.30	106.01	275.90	2.28	9.30	197.92
P3	2.12	7.03	2.54	1.96	3.28	63.93	214.22	2.12	5.04	153.33
T1	5.38	22.01	1.74	1.26	2.62	117.49	297.79	2.03	10.59	213.66
T2	3.63	19.42	1.70	1.52	2.42	111.53	281.38	2.05	8.54	201.84
Maximum value	5.38	22.01	2.54	1.96	3.89	117.49	297.79	2.18	10.59	213.66
Minimum value	1.86	7.03	1.53	1.26	2.42	57.61	143.37	1.65	7.37	102.97
Average value	2.99	15.62	2.05	1.66	3.15	89.12	239.56	2.05	8.12	171.80

**Table 7:** Igeo values of the soil uranium and heavy metal accumulation index and evaluation results of the pollution level in a uranium mining area in northern Guangdong.

Sampling Points		Heavy Metal Elements									I_tot_
		Mn	Co	Ni	Cu	Zn	As	Cd	Pb	U	
P0	*I*_geo_	0.72	3.20	0.59	0.28	1.38	5.70	7.23	0.54	2.30	21.93
	Grade	1	4	1	1	2	6	6	1	3	6
P1	*I*_geo_	0.31	2.82	0.02	0.08	1.18	5.26	6.58	0.14	2.40	18.80
	Grade	1	3	1	1	2	6	6	1	3	6
P2	*I*_geo_	0.71	3.80	0.75	0.28	1.14	6.14	7.52	0.61	2.63	23.60
	Grade	1	4	1	1	2	6	6	1	2	6
P3	*I*_geo_	0.50	2.23	0.76	0.39	1.13	5.41	7.16	0.50	1.75	19.82
	Grade	1	3	1	1	2	6	6	1	2	6
T1	*I*_geo_	1.84	3.88	0.21	−0.25	0.80	6.29	7.63	0.44	2.82	23.66
	Grade	2	4	1	0	1	6	6	1	3	6
T2	*I*_geo_	1.27	3.69	0.18	0.02	0.69	6.22	7.55	0.45	2.51	22.58
	Grade	2	4	1	1	1	6	6	1	3	6
Average	*I*_geo_	0.89	3.27	0.42	0.13	1.05	5.84	7.28	0.44	2.40	20.56

**Table 8:** Statistical table of ecological risk assessment of soil uranium and heavy metals in a uranium mining area in northern Guangdong.

Sampling Points	*E_r_^i^*									*RI*	Pollution Level
	Mn	Co	Ni	Cu	Zn	As	Cd	Pb	U		
P0	2.48	68.81	11.31	9.08	3.89	781.40	6741.18	10.90	220.97	7850.02	Very High Risk
P1	1.86	52.93	7.63	7.95	3.41	576.08	4301.03	8.24	236.78	5195.91	Very High Risk
P2	2.45	104.71	12.65	9.14	3.30	1175.29	8276.91	11.42	133.09	9728.96	Very High Risk
P3	2.12	97.00	12.69	9.81	3.28	1060.20	6426.62	10.58	151.19	7773.49	Very High Risk
T1	5.38	63.54	8.68	6.29	2.62	642.50	8933.82	10.15	270.92	9943.90	Very High Risk
T2	3.63	35.11	8.48	7.58	2.42	639.11	8441.47	10.24	256.09	9404.13	Very High Risk
Average	2.99	78.12	10.24	8.31	3.15	891.16	7186.84	10.25	243.62	8434.68	Very High Risk
Pollution level	No Risk	Low Risk	No Risk	No Risk	No Risk	High Risk	Very High Risk	No Risk	High Risk	Very High Risk	-

Note: “-” means not specified.

**Table 9:** Mortality results from ecotoxicity tests (%).

Genus’s Species	Time (h)	P0	P1	P2	P3	T1	T2
*C. vidua*	24	0	0	6.7	6.7	0	13.3
	48	0	6.7	6.7	6.7	6.7	13.3
	72	3.3	6.7	6.7	6.7	6.7	20
	96	10.0	6.7	20	13.3	20	23.3
*H.* sp.	24	3.3	0	6.7	0	10	0
	48	6.7	6.7	20	0	16.7	3.3
	72	10	10	36.7	0	36.7	13.3
	96	16.7	10	43.3	3.3	40	23.3

## Data Availability

Due to the nature of this research, participants of this study did not agree for their data to be shared publicly, so supporting data is not available.
